# Targeted radiosensitisation by pegylated liposome-encapsulated 3′, 5′-O-dipalmitoyl 5-iodo-2′-deoxyuridine in a head and neck cancer xenograft model

**DOI:** 10.1038/sj.bjc.6601958

**Published:** 2004-06-15

**Authors:** K J Harrington, K N Syrigos, P S Uster, A Zetter, C R Lewanski, W J Gullick, R G Vile, J S W Stewart

**Affiliations:** 1 ICRF Oncology Unit, Imperial College of Science, Technology and Medicine, Hammersmith Hospital, London W12 0HS, UK; 2 Cancer Research UK Targeted Therapy Laboratory, Chester Beatty Laboratories, Institute of Cancer Research, 237 Fulham Road, London SW3 6JB, UK; 3 SEQUUS Pharmaceuticals Inc., Menlo Park, CA 94025, USA; 4 Molecular Medicine Program, Mayo Clinic, Rochester, MN 55902, USA

**Keywords:** 5-iodo-2′-deoxyuridine, pegylated liposome, prodrug, radiosensitiser

## Abstract

5-Iodo-2′-deoxyuridine (IUdR) is an effective radiosensitiser but its clinical development has been limited by toxicity. Prolonged intravenous infusions of IUdR are necessary for optimal tumour uptake but cause dose-limiting myelosuppression. The lack of selective tumour uptake can lead to radiosensitisation of adjacent normal tissues and enhanced local radiation toxicity. Liposomal IUdR delivery offers selective targeting of tumour tissues and avoidance of local and systemic toxicity. In these studies, we report the development of a pegylated liposome containing a lipophilic IUdR derivative (3′, 5′-O-dipalmitoyl-5-iodo-2′-deoxyuridine) for use in a head and neck cancer xenograft model. Initial studies confirmed the ability of IUdR to sensitise two head and neck cancer cell lines to single fractions of radiotherapy (SFRT) and this effect was seen to correlate with the thymidine replacement index in KB cells. *In vivo* delivery of single doses of either unencapsulated IUdR or pegylated liposomal IUdR (PLIUdR) to nude mice bearing KB xenograft tumours did not enhance the effect of SFRT delivered 16 h later. When PLIUdR was delivered by a protracted administration schedule to a dose of 48 mg kg^−1^ over 7 days, it enhanced the effect of both 4.5 Gy SFRT and fractionated radiotherapy. PLIUdR was at least as effective as unencapsulated IUdR delivered by multiple intravenous injections or continuous subcutaneous infusion. Immunohistochemistry with a specific anti-IUdR monoclonal antibody confirmed greater levels of tumour staining in tumours from animals treated with PLIUdR compared with those treated with unencapsulated IUdR.

Halogenated pyrimidines (HP) (5-bromo-2′-deoxyuridine (BUdR) and 5-iodo-2′-deoxyuridine (IUdR)) are effective radiosensitisers in cells that take them up (Fowler and Kinsella, 1996). They act by being incorporated into DNA in competition with thymidine during S phase of the cell cycle and mediate radiosensitisation by increasing the susceptibility of IUdR- or BUdR-substituted DNA to radiation-generated reactive free radicals, which may also damage unsubstituted complementary-strand DNA (Fornace *et al*, 1990). In addition, there is evidence that HP can inhibit the repair of radiation-induced DNA damage (Iliakis *et al*, 1989). Exposure to clinically achievable steady-state concentrations of either IUdR or BUdR significantly increases formation of single- and double-stranded DNA breaks *in vitro* (Kinsella *et al*, 1987). HP-mediated radiosensitisation is directly related to the extent of thymidine replacement in DNA (thymidine replacement index, TRI) (Lawrence *et al*, 1990; Miller *et al*, 1992), which, in turn, is related to the duration of drug exposure *in vitro* (Lawrence *et al*, 1990). Phase I clinical studies using protracted intravenous infusions of IUdR in patients with hepatic metastases, high-grade glioma, sarcomas and head and neck cancers resulted in TRI of 4–26% in tumour tissue, which were significantly greater than the levels in adjacent normal tissues (Speth *et al*, 1988; Cook *et al*, 1992). Haematological and mucosal toxicities (including exacerbation of the local radiation response) were dose-limiting and directly related to the duration of drug infusion (Urtasun *et al*, 1993; Epstein *et al*, 1994; Sullivan *et al*, 1994). These findings may, in part, explain the failure of intravenous infusional IUdR to yield a survival advantage when used as a radiosensitiser in the treatment of gliomas, head and neck cancers and sarcomas (Goffman *et al*, 1992; Urtasun *et al*, 1993; Epstein *et al*, 1994; Sullivan *et al*, 1994; Robertson *et al*, 1995; Urtasun *et al*, 1996). *In vitro* data have suggested that repeated short duration drug exposures may provide a means of achieving effective DNA incorporation and reducing dose-limiting myelotoxicity (Lawrence *et al*, 1990), but relatively few cells will be targeted with this approach since only cells in S phase at the time of drug administration will incorporate the agent. Fowler and Kinsella (1996) have calculated that infusion durations of 5–10 times greater than the population doubling time of clonogenic cells will be required for clinically significant radiosensitisation. Therefore, although HP appear to be a promising group of agents, their efficacy is limited by the amount of drug incorporated into the tumour with relatively short durations of drug infusion and the occurrence of dose-limiting systemic toxicity when more prolonged infusions are administered.

One potential means of overcoming both of these obstacles is the development of liposome-targeted HP. The inclusion of methoxypolyethylene glycol-derivatised (pegylated) lipids in the bilayer membrane of liposomes effectively increases the longevity of liposomes in the circulation (Papahadjopoulos *et al*, 1991) and increases their accumulation in tumours (Huang *et al*, 1992). Preclinical studies have shown that cytotoxic drugs entrapped in pegylated liposomes are active against a range of tumours (Harrington *et al*, 2000a). Clinical studies of pegylated liposomal doxorubicin have confirmed its activity against AIDS-related Kaposi's sarcoma (Northfelt *et al*, 1998; Stewart *et al*, 1998) and breast and ovarian cancers (Muggia *et al*, 1997; Ranson *et al*, 1997) with considerable attenuation of the adverse effects of the unencapsulated drug. Furthermore, preclinical and Phase I/II trials of pegylated liposomal doxorubicin and radiotherapy (RT) have supported the feasibility of this approach in head and neck and lung cancers (Koukourakis *et al*, 1999; Harrington *et al*, 2000b, 2000c; Harrington *et al*, 2001a). In the present study, we carried out experiments to examine the potential value of a novel prodrug formulation of IUdR (3′, 5′-O-dipalmitoyl 5-iodo-2′-deoxyuridine, dpIUdR) encapsulated in pegylated liposomes (PLIUdR) to act as a radiation sensitiser in a human head and neck squamous cell cancer (HNSCC) model.

## MATERIALS AND METHODS

### *In vitro* radiosensitivity assay

Human HNSCC KB and HN5 cells were grown as monolayers in 75 cm^2^ tissue culture flasks (Falcon, NJ, USA) in Dulbecco's modified Eagle's medium supplemented with 10% fetal calf serum (FCS) (Gibco, Paisley, UK), nonessential amino acids, and antibiotics at 37°C in a humidified incubator with 5% CO_2_. Culture medium was supplied by the Media Production Unit at the Imperial Cancer Research Fund, Clare Hall, Herts, UK. Under these conditions, the doubling time of KB cells is approximately 17 h (data not shown). The cells were plated at low density (5 × 10^4^ cells flask^−1^) to ensure that they were growing exponentially during the 48 h period in which they were exposed to 0, 10^−6^, 10^−5^ or 10^−4^ M unencapsulated IUdR (Nova Laboratories, Leicester, UK). For the assessment of radiation survival after exposure to IUdR, the cells were trypsinised and plated in appropriate dilutions in growth medium in six-well plates. At 4 h after plating, the cells were irradiated (0, 4, 7 Gy) with a 111 TBq ^137^Cs source (CIS Bio International, Gif-sur-Yvette, France) yielding a dose rate of about 2 Gy min^−1^. Following irradiation, the cells were incubated for 6 days, changing the culture medium on alternate days after day 3. After this time, surviving cells were trypsinised and plated onto 96-well microtitre plates containing 100 *μ*l of medium per well. After incubation overnight, growth medium was removed and the wells were washed twice with phosphate-buffered saline (PBS) and 60 *μ*l of 3.75 mM *p*-nitrophenyl-*N*-acetyl-*β*-D-glucosaminide (NAG) in 0.05 M citrate buffer (0.1 M citric acid, 0.1 M trisodium citrate, 0.25% Triton X-100, pH 5.0) was added to each well (Yagi *et al*, 1989). Cells were incubated at 37°C and 5% CO_2_ for 1 h, removed from the incubator and the colorimetric reaction was stopped by the addition of 90 *μ*l of glycine buffer (50 mM glycine, 5 mM EDTA, pH 10.4). The optical density was read on a Titertek Multiskan® MCC/340 spectrophotometer with a 405 nm filter.

### Thymidine replacement index

KB cells were grown as monolayers in 75 cm^2^ tissue culture flasks as detailed above. The cells were plated at low density (5 × 10^4^ cells flask^−1^) to ensure that they were growing exponentially during the 48 h period in which they were exposed to 0, 10^−6^, 10^−5^ or 10^−4^ M unencapsulated IUdR. After this time, the cells were trypsinised, centrifuged and the pellet was washed thrice with PBS and the DNA was extracted using a QIAamp Tissue Kit (Qiagen Ltd, Sussex, UK) according to the manufacturer's instructions. Digestion of DNA into deoxynucleotides was performed as follows: 100 *μ*l of the DNA preparation (containing 5–10 *μ*g DNA) was incubated for 1 h at 37°C with 0.4–0.5 *μ*l DNase I (Boehringer Mannheim, Germany) and 5 *μ*l 100 mM MgCl_2_; 0.4 *μ*l of crotalus venom phosphodiesterase I (Boehringer Mannheim) was added and incubated for 1 h at 37°C; 0.2 *μ*l alkaline phosphatase (Boehringer Mannheim) was added and incubated for 1 h at 37°C. The resulting nucleotide solution was adjusted to pH 5.5 before loading onto the HPLC column. Standard nucleotides (2′-deoxycytidine [C], 2′-deoxyguanosine [G], 2′-deoxyadenosine [A] and thymidine [T]) were obtained from Sigma Chemical Co. (St Louis, MO, USA). All HPLC experiments were performed using a C18 Phenomenex Columbus column (2 × 250 mm, 5 *μ*m particle size) with UV detection at 254 and 288 nm. The mobile phase contained 10 mM ammonium acetate, pH 6.0, with 8.75% acetonitrile, pumped at a flow rate of 1 ml min^−1^ at room temperature. The deoxynucleotides and IUdR were detected at 254 and 288 nm. To calibrate the peaks on the HPLC, standard stock solutions of IUdR and deoxynucleotides were diluted to yield several solutions with concentrations of 2 × 10^−4^ to 2 × 10^−6^ M and a linear relationship was observed between the peak heights and amounts loaded (data not shown).

### Tumour xenograft model

All animal experiments were carried out with ethical committee approval and met the standards laid out in the UKCCCR guidelines (Workman *et al*, 1998). Female nude mice of mixed genetic background bred under specific pathogen-free conditions at the Imperial Cancer Research Fund Animal Breeding Unit (South Mimms, Herts, UK) were used. Animals were housed in sterile filter-top cages on sterile bedding and maintained on an irradiated diet and autoclaved acidified water (pH 2.8) *ad libitum*. KB tumour cells were grown to confluence *in vitro* in 175 cm^2^ tissue culture flasks as detailed above. Cells were harvested and a single cell suspension was prepared. Tumour xenografts were set up by subcutaneous injection of 5 × 10^6^ tumour cells in 100 *μ*l of culture medium into the right flank of the mice. The animals were used for experiments approximately 15 days later, at which time the tumours were 6–8 mm in diameter. Starting 7 days after inoculation the tumours were measured on alternate days on at least three occasions before the commencement of the study. Three orthogonal tumour diameters were recorded using Vernier calipers and the tumour diameter was calculated from the formula: 
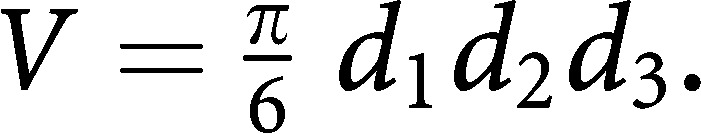
. Therapeutic irradiation (see below) was administered on day 15 after inoculation and the measured tumour volume on this day was designated as the initial volume or *V*_0_. Subsequently, the tumour volume was assessed two or three times per week and the absolute and relative (as compared to *V*_0_) tumour volume was calculated. Mice were killed after the tumour had increased in size to more than three times its original volume (3*V*_0_). The time taken to reach 3*V*_0_ was recorded and used as a surrogate measure of animal survival and was designed to spare the animals from the physical distress of unnecessarily large tumour burdens and to comply with the UKCCCR guidelines (Workman *et al*, 1998).

### 5-Iodo-2′-deoxyuridine for *in vivo* use

Unencapsulated 5-iodo-2′-deoxyuridine (Sigma-Aldrich Co. Ltd., Poole, UK) was dissolved in a 20% solution of dimethyl sulphoxide in distilled water. Because of its water solubility and low molecular weight, IUdR cannot be retained efficiently in liposomes. Therefore, IUdR was derivatised by attaching two long-chain fatty acids (palmitic acid) to the 3′ and 5′ positions of the IUdR sugar moiety to form 3′, 5′-O-dipalmitoyl-5-iodo-2′-deoxyuridine (dpIUdR) to convert the agent into a hydrophobic, lipid soluble prodrug which was stably incorporated into the bilayer of pegylated liposomes with the following lipid composition (values expressed as a % molar ratio): HSPC (94.6%) and MPEG-DSPE (5.4%). The agent was supplied as an isotonic, preservative-free solution in 20 ml vials at a dpIUdR concentration of between 2.8 and 3.5 mg ml^−1^ (equivalent to IUdR concentrations of 1.2–1.5 mg ml^−1^).

### Administration of test agents

Unencapsulated IUdR was administered either as intravenous bolus injections or as a continuous subcutaneous infusion. The intravenous bolus injections were administered according to the following schedules: (1) single injections of 24 mg kg^−1^ given 16 h before tumour irradiation; (2) as a series of four injections of 12 mg kg^−1^ on alternate days to a total dose of 48 mg kg^−1^ over a 7-day period before irradiation. The continuous infusions of unencapsulated IUdR were administered using subcutaneously implanted Alzet® Model 2002 mini-osmotic pumps (Charles River UK, Ltd, Kent, UK) over a 7-day period before irradiation. A dose of 48 mg kg^−1^ was delivered over the 7-day period (6.9 mg kg day^−1^) by pumps with a mean fill volume of 226±9 *μ*l and a mean pumping rate of 0.46±0.03 *μ*l h. PLIUdR was administered as intravenous bolus injections according to the following protocols: (1) single injections of 24 mg kg^−1^ given 16 h before tumour irradiation; (2) as a series of four injections of 12 mg kg^−1^ to a total dose of 48 mg kg^−1^ over a 7-day period before irradiation. No attempt was made to deliver subcutaneous infusions of PLIUdR.

### Tumour irradiation

Tumour irradiation was performed using the ^137^Cs source with mice carefully positioned within a specially constructed jig as described previously (Harrington *et al*, 2000c). The system was calibrated as previously described (Harrington *et al*, 2000c) with lithium fluoride thermoluminescent dosimeters (TLD) (Nuclear Enterprises, Reading, UK) that were read in a Toledo 654 TLD reader (DA Pitman, Weybridge, UK) (data not shown). Before therapeutic irradiation, the animals were anaesthetised with an intraperitoneal injection of 100 *μ*l of a 1 : 1 : 4 mixture of Hypnorm (fentanyl citrate 0.315 mg ml^−1^, fluanisone 10 mg ml^−1^) (Janssen-Cilag Ltd, High Wycombe, UK), Hypnovel® (midazolam 5 mg ml^−1^) (Roche Products Ltd, Welwyn Garden City, UK) and water for injection BP (Fresenius Health Care Group, Basingstoke, UK). Anaesthetised animals were positioned such that the subcutaneous xenograft tumours were exposed to the radiation beam with the rest of the animals’ bodies shielded by 4 cm thick lead. Great care was taken to avoid direct pressure on the tumour mass in order to minimise the risk of creating areas of pressure-induced hypoxia during irradiation. Animals were managed at all times in accordance with UKCCCR standards (Workman *et al*, 1998).

### Radiotherapy alone

The effect of single fractions of RT (SFRT) was initially assessed by irradiating groups of mice bearing KB xenograft tumours with radiation doses of 4.5 Gy (*n*=17) and 9 Gy (*n*=12) over a period of 385 and 770 s, respectively. The RT was delivered at a dose rate of 0.7 Gy min^−1^ as determined by the dosimetric calibration outlined above. Similarly, the effect of daily fractionated RT (FRT) to a dose of either 9 Gy in three fractions over 3 days (*n*=11) or 15 Gy in five fractions over 5 days (*n*=10) was determined in KB tumour-bearing mice. Again, all irradiations were carried out at a dose rate of 0.7 Gy min^−1^ over a period of 257 s.

### Radiotherapy and IUdR

The combination of RT and IUdR was tested according to a number of sequential protocols with the aim of modelling the clinical use of IUdR as a radiation sensitiser in patients with solid cancers. In the initial studies, tumour-bearing mice received intravenous bolus doses of 24 mg kg^−1^ of IUdR, either in the form of unencapsulated IUdR or PLIUdR, 16 h before receiving SFRT at doses of either 4.5 or 9 Gy. In subsequent studies, an attempt was made to model the clinical experience with IUdR in which prolonged intravenous infusions of the drug have been administered as loading doses before RT. Therefore, four separate intravenous bolus doses of 12 mg kg^−1^ of either unencapsulated IUdR or PLIUdR were administered on alternate days over a 7-day period (−8 to −1 days) before SFRT was delivered to a dose of either 4.5 or 9 Gy. Following on from the studies using SFRT, a number of studies of FRT were performed in which IUdR was administered according to the protracted loading schedule. Tumour-bearing mice received total doses of 48 mg kg^−1^ of IUdR, either as four doses of 12 mg kg^−1^ of unencapsulated IUdR or PLIUdR or as a subcutaneous infusion of unencapsulated IUdR administered over a 7-day period as described above. FRT was administered to doses of either 9 Gy in three fractions over 3 days or 15 Gy in five fractions over 5 days.

### Immunohistochemistry

The monoclonal anti-BUdR antibody (clone BU-1), which recognises both IUdR and BUdR, was used (Amersham International plc, Amersham, UK). KB tumour-bearing animals (three for each treatment) received total doses of 48 mg kg^−1^ of IUdR, either as four doses of 12 mg kg^−1^ of PLIUdR or as a subcutaneous infusion of unencapsulated IUdR administered via Alzet® Model 2002 miniosmotic pumps over a 7-day period as described above. Control animals received four bolus injections of PBS over the same time course as that used for PLIUdR. The tumours were removed 24 h after completion of IUdR administration and fixed in formalin at 4°C for 3 days. Thereafter, they were embedded in paraffin and 5 *μ*m sections were cut and mounted on slides coated with 0.1% poly-L-lysine (Sigma, Poole, UK), dried overnight and stored at room temperature until use. The slides were dewaxed and rehydrated through solutions of xylene, alcohol and water. They were then incubated overnight at 4°C with 50 *μ*l of BU-1 antibody that had previously been biotinylated (3–4 molecules per molecule of antibody). After this incubation, streptavidin–horseradish peroxidase (Dako Ltd, High Wycombe, UK) was added and subsequent colorimetric visualisation was achieved by addition of 50 ml 3,3′-diaminobenzidine (BDH Laboratory Supplies, Poole, UK) solution at a concentration of 0.3 mg ml^−1^.

### Toxicity assessment

Serial measurements of animal weight were used as a surrogate measure of systemic toxicity. The cutaneous radiation reaction in the treated area was observed in all treatment groups. No attempt was made to measure haematological or biochemical parameters.

### Statistical analysis

Relative tumour volumes were recorded for each animal in a treatment group. The times taken to reach 3*V*_0_ were recorded as an indication of the progression of the xenograft tumours as described above. The Wilcoxon rank-sum test was used to test the difference between the times taken to reach 3*V*_0_ in the various test groups. Differences were considered to be significant at *P*<0.05.

## RESULTS

### *In vitro* radiation response of SCCHN cells

**Figure 1:**
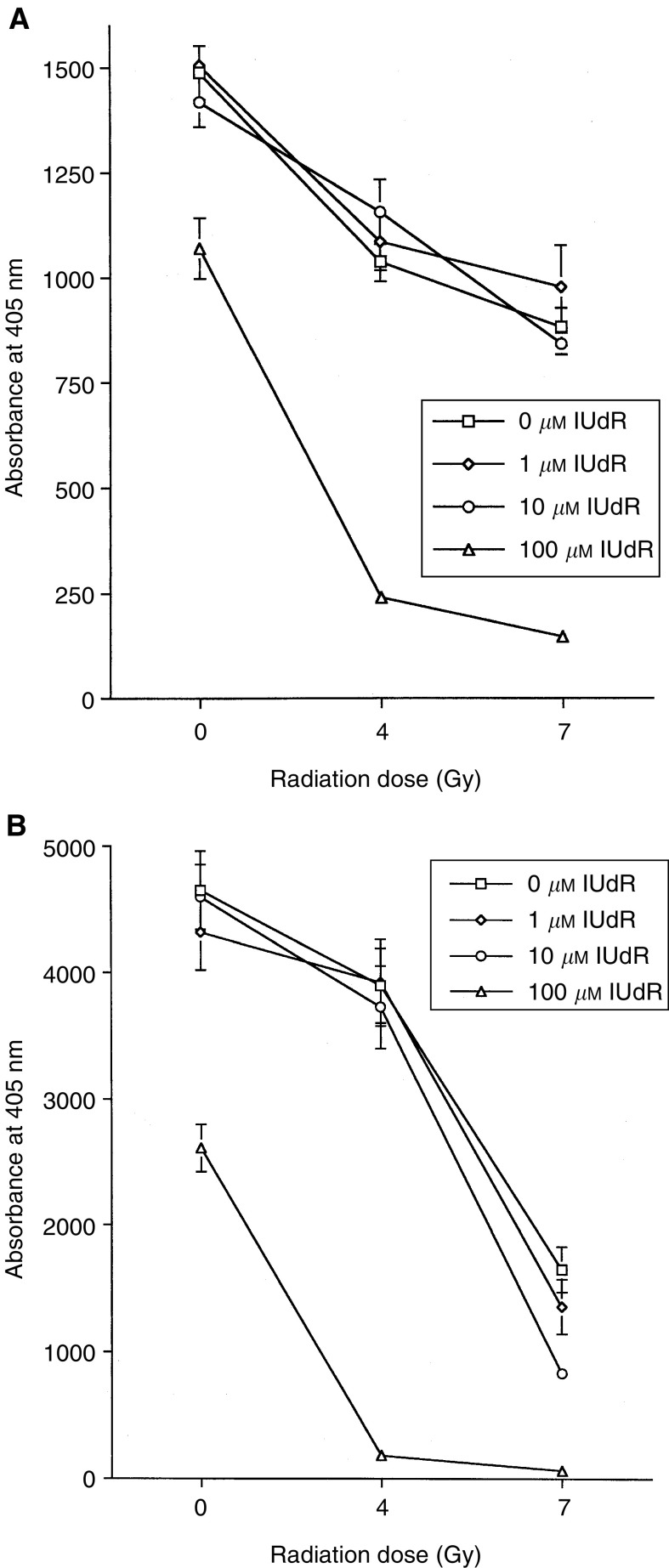
Effect of IUdR treatment on the response of SCCHN cells to irradiation: (**A**) HN5 cells; (**B**) KB cells. Cells were exposed to 0, 1, 10 or 100 *μ*M unencapsulated IUdR for 48 h and then irradiated (0, 4 or 7 Gy) with a ^137^Cs source at a dose rate of 2 Gy min^−1^. Following irradiation, cells were incubated for 6 days and a *p*-nitrophenyl-*N*-acetyl-*β*-D-glucosaminide (NAG) cell survival assay was performed. Significant radiosensitisation was seen at the 100 *μ*M concentration for both the 4 and 7 Gy radiation doses in each cell line. The effect of IUdR was most pronounced for the KB cells and this cell line was selected for subsequent *in vitro* and *in vivo* analysis.

The effect of unencapsulated IUdR at concentrations between 10^−4^ and 10^−6^ M on the survival of HN5 and KB cells after irradiation is presented in Figure 1A and B

. Significant radiosensitisation was seen at the 10^−4^ M concentration for both the 4 and 7 Gy radiation doses in each cell line. This effect was more pronounced in the KB cells (Figure 1B) and this cell line was used for subsequent experiments.

### TRI after *in vitro* exposure of KB tumour cells

The retention times for C, G, T, A and IUdR, were 4.6±0.4, 5.0±0.4, 6.2±0.2, 7.3±0.2 and 10.8±0.2 min, respectively. IUdR was preferentially detected at 288 nm and the deoxynucleotides at 254 nm. The compositions of all the deoxynucleotides showed good agreement between control and treated cells, with the exception of thymidine that was partially replaced by IUdR in treated cells. The percentage substitution by IUdR was equal to the percentage by which thymidine was decreased from the control cells, suggesting that thymidine was substituted by IUdR without any other effect on the base content of the DNA (Table 1

**Table 1:** Thymidine replacement indices (TRI) for KB cells after 48 h period of exposure to unencapulated IUdR at concentrations between 0 and 100 *μ*M

**IUdR concentration (*μ*M)**	**TRI**	**Thymidine content in DNA (%)**
0	0	29.8
1	2.2	27.6
10	2.8	27
100	7	22.8

).

### SFRT and single bolus IUdR

The results of SFRT at doses of 4.5 and 9 Gy delivered 16 h after a single bolus injection of 24 mg kg^−1^ of either unencapsulated IUdR or PLIUdR are presented in Table 2

**Table 2:** Effect of single dose unencapsulated IUdR or PLIUdR 24 mg kg^−1^ plus single fraction RT against KB xenograft tumours in nude mice

	**Median time to 3*V*_0_ (days)**	
**Group**	**4.5 Gy**	**9 Gy**
No drug	12.7	22.6
Unencapsulated IUdR plus RT	14.0	21.9
PLIUdR plus RT	12.4	22.7
		
	***P***	
**Group**	**4.5 Gy**	**9 Gy**
RT *vs* unencapsulated IUdR plus RT	>0.1	>0.1
RT *vs* PLIUdR plus RT	>0.1	>0.1
Unencapsulated IUdR plus RT *vs* PLIUdR plus RT	>0.1	>0.1

Median times to 3*V*_0_ and statistical analysis.

. As compared to the untreated controls, neither unencapsulated IUdR nor PLIUdR alone exerted a significant effect on tumour growth. In combination with SFRT doses of 4.5 and 9 Gy, there was no evidence of enhancement of the radiation response with either agent (data not shown). These results prompted attempts to increase the area under the curve of tumour exposure to IUdR by using more protracted dosing schedules.

### SFRT and protracted administration of IUdR

The effect of repeated bolus administration of unencapsulated IUdR and PLIUdR (total dose 48 mg kg^−1^ over 7 days) before SFRT at doses of either 4.5 and 9 Gy on the growth of KB xenograft tumours is shown in Table 3

**Table 3:** Effect of multiple dose unencapsulated IUdR or PLIUdR (4 × 12 mg kg^−1^) plus single fraction RT against KB xenograft tumours in nude mice

	**Median time to 3*V*_0_ (days)**	
**Group**	**4.5 Gy**	**9 Gy**
No drug	12.7	22.6
Unencapsulated IUdR plus RT	13.7	24.3
PLIUdR plus RT	16.8	26.7
	***P***	
**Group**	**4.5 Gy**	**9 Gy**
RT *vs* unencapsulated IUdR plus RT	>0.1	>0.1
RT *vs* PLIUdR plus RT	<0.01	>0.1
Unencapsulated IUdR plus RT *vs* PLIUdR plus RT	>0.1	>0.1

Median times to 3*V*_0_ and statistical analyses.

. Once again, neither of these agents exerted a significant independent effect on tumour growth (data not shown). When administered according to this protracted schedule, PLIUdR significantly increased the effect of SFRT at a dose of 4.5 Gy (*P*<0.01) but not at a dose of 9 Gy (*P*>0.1). There was no evidence of enhancement of the effect of SFRT at either dose level with unencapsulated IUdR (*P*>0.1). Furthermore, a direct comparison between the effects of PLIUdR and unencapsulated IUdR revealed no significant difference at 4.5 or 9 Gy (*P*>0.1 for both comparisons). There was no evidence of increased local cutaneous radiation toxicity with unencapsulated IUdR or PLIUdR at either radiation dose.

### FRT and protracted administration of IUdR

The effect of FRT to a dose of either 9 Gy in three fractions in 3 days (9 Gy in 3F) or 15 Gy in five fractions in 5 days (15 Gy in 5F) in combination with either a subcutaneous infusion of 48 mg kg^−1^ of unencapsulated IUdR or four bolus injections of 12 mg kg^−1^ of PLIUdR over 7 days on the growth of KB tumour xenografts is shown in Table 4

**Table 4:** Effect of a subcutaneous infusion of unencapsulated IUdR (48 mg kg^−1^) or multiple dose PLIUdR (4 × 12 mg kg^−1^) plus fractionated RT on KB xenograft tumours in nude mice

	**Median time to 3*V*_0_ (days)**	
**Group**	**9 Gy in 3F**	**15 Gy in 5F**
RT alone	13.2	18.8
sc unencapsulated IUdR 48 mg kg^−1^ plus RT	17.6	26.9
PLIUdR 4 × 12 mg kg^−1^ plus RT	21.6	34.2
	***P***	
**Group**	**9 Gy in 3F**	**15 Gy in 5F**
RT *vs* sc unencapsulated IUdR plus RT	>0.1	0.1>P>0.05
RT *vs* PLIUdR plus RT	<0.05	<0.01
sc unencapsulated IUdR plus RT *vs* PLIUdR plus RT	>0.1	>0.1

**Figure 2:**
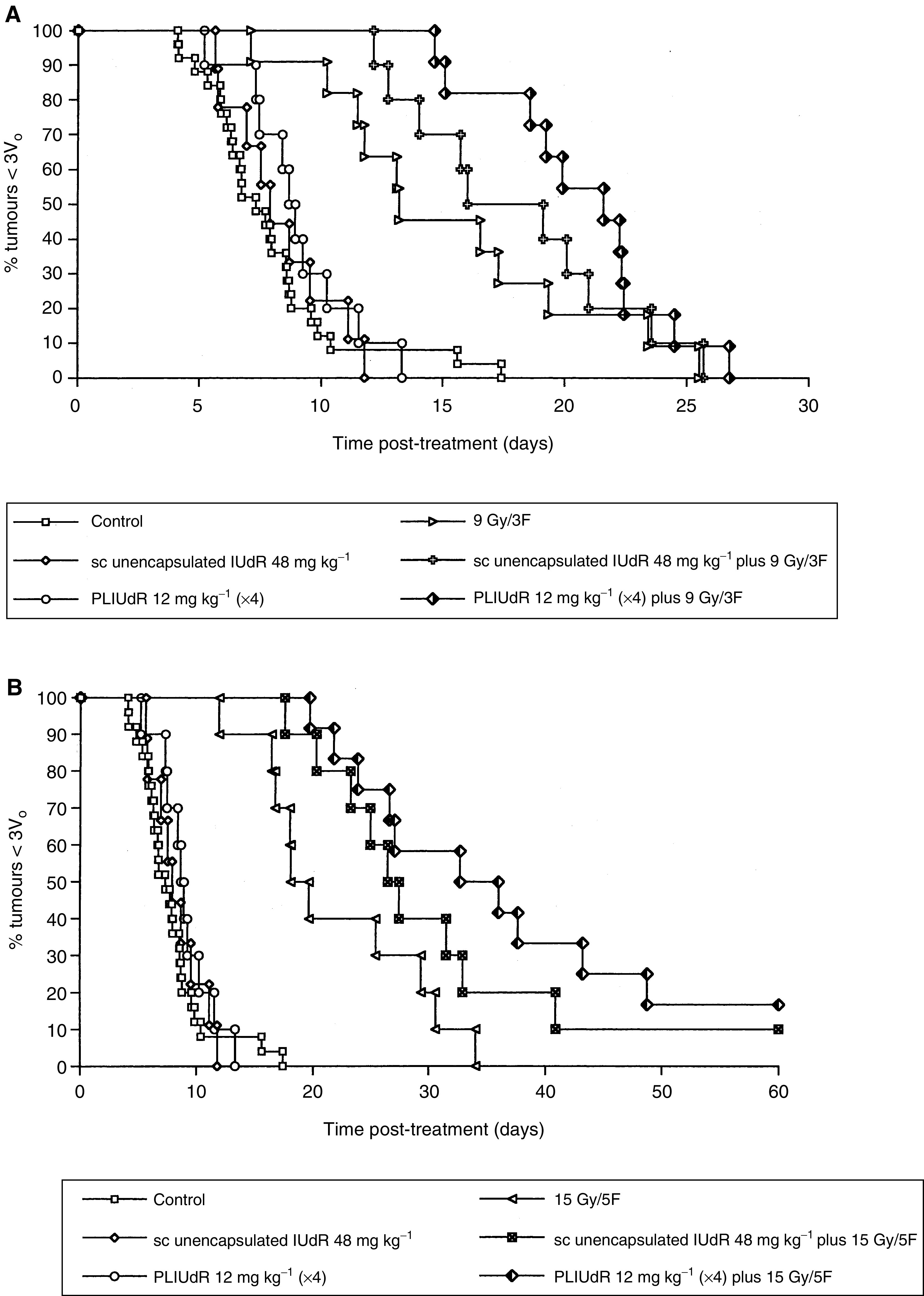
Effect of a subcutaneous infusion of unencapsulated IUdR (48 mg kg^−1^) or four intravenous doses of 12 mg kg^−1^ PLIUdR (total dose 48 mg kg^−1^) on the response of KB xenograft tumours to fractionated irradiation: (**A**) 9 Gy in three fractions over 3 days; (**B**) 15 Gy in five fractions over 5 days. Test agents were administered over a period of 7 days terminating 16 h before the delivery of the first dose of FRT. PLIUdR administered according to this protracted schedule enhanced the effect of both FRT schedules (*P*<0.05 for 9 Gy in three fractions; *P*<0.01 for 15 Gy in five fractions). The effect of unencapsulated IUdR did not reach statistical significance.

Median times to 3*V*_0_ and statistical analyses.

and in Figure 2A and B

. When administered according to this schedule, PLIUdR significantly increased the effect of FRT at doses of both 9 Gy in 3F (*P*<0.05) and 15 Gy in 5F Gy (*P*<0.01). However, the subcutaneous infusion of unencapsulated IUdR did not significantly increase the effect of either dose of FRT, although at the dose of 15 Gy in 5F it was of borderline significance (0.1>*P*>0.05). A direct comparison between the effects of multiple dose PLIUdR and subcutaneous unencapsulated IUdR revealed no significant difference at either dose of FRT (*P*>0.1 for both comparisons). There was no evidence of increased local radiation toxicity with subcutaneous unencapsulated IUdR or PLIUdR at either FRT dose.

### Immunohistochemistry

**Figure 3:**
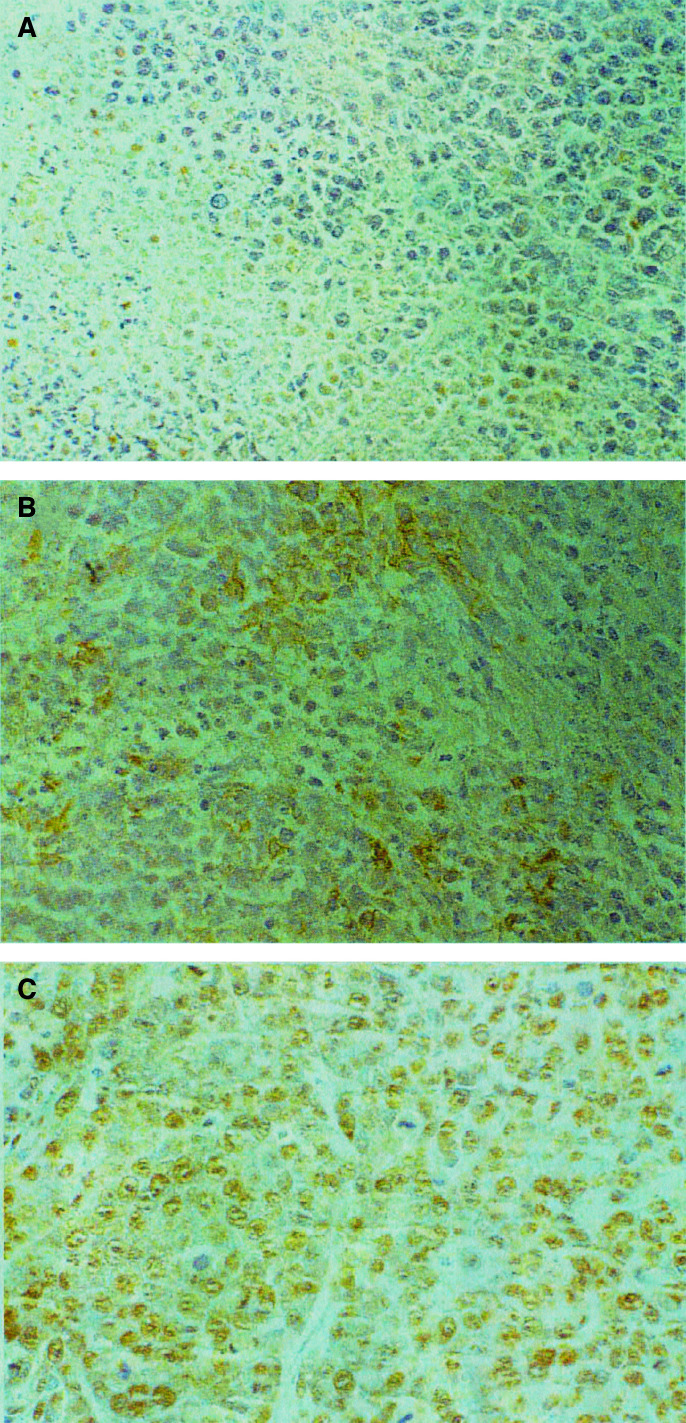
Immunohistochemistry of KB tumours from animals treated with (**A**) intravenous phosphate-buffered saline (four doses of 100 *μ*l over 7 days); (**B**) unencapsulated IUdR 48 mg kg^−1^ as a continuous subcutaneous infusion over 7 days; (**C**) intravenous PLIUdR (four doses of 12 mg kg^−1^ over 7 days). Paraffin-embedded sections were stained with biotinylated BU-1 monoclonal antibody followed by incubation with streptavidin–horseradish peroxidase and addition of 3,3′-diaminobenzidine. No positive staining was seen in the phosphate-buffered saline-treated group. The animals treated with unencapsulated IUdR showed low levels of staining. There was very prominent nuclear staining in the animals treated with PLIUdR.

The results of staining tumour specimens from animals treated with PBS, unencapsulated IUdR and PLIUdR with BU-1 monoclonal antibody are presented in Figure 3A, B and C

, respectively. Significant positive staining of the nuclei of tumour cells was seen in animals treated with PLIUdR and, to a lesser extent, unencapsulated IUdR. In contrast, there was virtually no tumour staining seen in the animals treated with PBS.

### Toxicity

There was no significant alteration in the weight of mice that received single intravenous bolus doses of 24 mg kg^−1^ of either unencapsulated IUdR or PLIUdR, SFRT or combinations of these two treatments (data not shown). However, multiple intravenous injections of either unencapsulated IUdR or PLIUdR were associated with a temporary reduction in the animals’ weights (<10%) over the week during which the injections were administered (data not shown). No such alteration was detected for the animals that had the subcutaneous infusion pumps inserted (data not shown). All groups that received FRT experienced a reversible 6–15% reduction in mean weight during the first 10 days of the study (data not shown). There was no evidence of enhanced cutaneous radiation reaction in any of the treatment groups (data not shown).

## DISCUSSION

In recent years, radiosensitising agents (cytotoxic drugs, hyperbaric oxygen, hypoxic cell sensitisers and HP) have been shown to improve the response rate and outcome of radical RT in a range of tumour types, including head and neck cancer (McGinn and Kinsella 1992; Munro 1995; Saunders and Dische 1996; Overgaard *et al*, 1998; Pignon *et al*, 2000). However, the combined use of RT and radiosensitising agents has a number of potential drawbacks: increased local normal tissue radiation toxicity that may necessitate radiation dose reductions and treatment delays (Tannock, 1996); increased late local radiation morbidity (Henk, 1997); and dose-limiting systemic toxicities. Liposome delivery offers the prospect of reducing or circumventing each of these problems by targeting delivery of the radiosensitiser preferentially to the tumour tissue and avoiding local and distant normal tissue deposition of the drug. We have recently provided proof of principle for this strategy using RT and liposomal doxorubicin and cisplatin in a KB tumour xenograft model (Harrington *et al*, 2000b). However, since each of those agents has been shown to have a demonstrable antitumour effect in this model (Harrington *et al*, 2000c), it was difficult to be clear that they were acting as true radiosensitisers.

In this study, we have demonstrated that a novel prodrug formulation of IUdR encapsulated within pegylated liposomes has no intrinsic antitumour efficacy but is able to enhance the effect of both SFRT and FRT in a HNSCC xenograft model in nude mice. Significantly, the liposomal agent was at least as effective as a sustained infusion of unencapsulated IUdR. However, it must be borne in mind that unencapsulated IUdR was not administered at the maximum tolerated dose in these experiments. Clearly, future studies will need to compare these two agents at their maximal tolerated doses in order to provide a more accurate picture of the radiosensitising effect of this liposomal prodrug formulation.

These data have considerable significance for the development of clinical strategies using pegylated liposomal radiosensitisers. In clinical practice, the conventional means of delivering IUdR has involved prolonged intravenous infusions over a period of up to 14 days before commencing RT (Speth *et al*, 1988; Cook *et al*, 1992; Goffman *et al*, 1992; Urtasun *et al*, 1993; Epstein *et al*, 1994; Sullivan *et al*, 1994; Robertson *et al*, 1995; Urtasun *et al*, 1996). Such schedules have been associated with appreciable systemic hematological toxicity and local catheter-related infusion site reactions and have effectively prevented the development of this promising agent for clinical use (Goffman *et al*, 1992; Urtasun *et al*, 1993; Sullivan *et al*, 1994; Epstein *et al*, 1994; Robertson *et al*, 1995; Urtasun *et al*, 1996). The data presented here suggest that administration of a small number of short-duration intravenous infusions of dpIUdR encapsulated in pegylated liposomes could achieve an equivalent radiosensitising effect as more prolonged intravenous infusions. Further studies will be required to determine if the liposomal agent is as active as protracted delivery of unencapsulated drug at the maximum tolerated dose. Although there was no direct assessment of toxicity in these studies, previous clinical experience with myelotoxic agents, such as doxorubicin, entrapped in pegylated liposomes has revealed attenuation of haematological toxicity (Muggia *et al*, 1997; Ranson *et al*, 1997).

The examination of the effect of various schedules of administration of unencapsulated IUdR and PLIUdR provides some useful insights into the use of these agents. Enhancement of the radiation response was not evident after single bolus injections of either agent delivered 16 h (approximately one *in vitro* doubling time) before SFRT at doses of 4.5 and 9 Gy. This lack of activity of PLIUdR may have been accentuated by relatively slow kinetics of conversion of the lipophilic prodrug to its hydrophilic active component and its subsequent diffusion into the interior of tumour cells to be incorporated into their DNA. However, when the IUdR was delivered according to a protracted administration schedule (approximately 10 *in vitro* doubling times), this resulted in enhanced efficacy of the SFRT and FRT doses. These findings are in keeping with the previously published preclinical and clinical data which suggest that for meaningful radiosensitisation to occur IUdR should be present for a prolonged period to ensure maximal labelling of tumour cells (Goffman *et al*, 1992; Urtasun *et al*, 1993; Epstein *et al*, 1994; Sullivan *et al*, 1994; Robertson *et al*, 1995; Fowler and Kinsella, 1996; Urtasun *et al*, 1996). The administration of unencapsulated IUdR by continuous subcutaneous infusion and PLIUdR by multiple bolus injections appeared to be an effective means of achieving this objective. Previous studies in nude mice bearing KB xenograft tumours have shown that ^111^In-DTPA-labelled pegylated liposomes have a prolonged circulation half-life of approximately 10 h and achieve maximal tumour uptake at 24 h after intravenous injection (Harrington *et al*, 2000d). Biodistribution and pharmacokinetic studies in patients with solid cancers have demonstrated similar data, with a circulation half-life of approximately 75 h and maximal tumour uptake at 48–72 h (Harrington *et al*, 2001b). Therefore, progressive accumulation of PLIUdR within tumour deposits may effectively act as a local, prolonged, high-concentration infusion of the agent precisely in the site where it is likely to exert maximum efficacy.

Therefore, in summary, this novel prodrug formulation of IUdR encapsulated in pegylated liposomes appears to offer considerable promise for further development. Indeed, preliminary pharmacokinetic and biodistribution studies have commenced in patients with head and neck cancer.
